# Family and species as determinants modulating mineral composition of selected wild-growing mushroom species

**DOI:** 10.1007/s11356-020-10508-6

**Published:** 2020-08-18

**Authors:** Mirosław Mleczek, Anna Budka, Pavel Kalač, Marek Siwulski, Przemysław Niedzielski

**Affiliations:** 1 Department of Chemistry, Poznan University of Life Sciences, Poznań, Poland; 2 Department of Mathematical and Statistical Methods, Poznan University of Life Sciences, Poznań, Poland; 3 Department of Applied Chemistry, Faculty of Agriculture, University of South Bohemia, České Budějovice, Czech Republic; 4 Department of Vegetable Crops, Poznan University of Life Sciences, Poznań, Poland; 5 Faculty of Chemistry, Adam Mickiewicz University in Poznań, Poznań, Poland

**Keywords:** Wild-growing mushrooms, Mineral elements, Mushroom family, Mushroom species, Accumulation

## Abstract

It has been known since the 1970s that differences exist in the profile of element content in wild-growing mushroom species, although knowledge of the role of mushroom species/families as determinants in the accumulation of diverse element remains limited. The aim of this study was to determine the content of 63 mineral elements, divided into six separate groups in the fruit bodies of 17 wild-growing mushroom species. The mushrooms, growing in widely ranging types of soil composition, were collected in Poland in 2018. *Lepista nuda* and *Paralepista gilva* contained not only the highest content of essential major (531 and 14,800 mg kg^−1^, respectively of Ca and P) and trace elements (425 and 66.3 mg kg^−1^, respectively of Fe and B) but also a high content of trace elements with a detrimental health effect (1.39 and 7.29 mg kg^−1^, respectively of Tl and Ba). A high content of several elements (Al, B, Ba, Bi, Ca, Er, Fe, Mg, Mo, P, Sc, Ti or V) in *L. nuda*, *Lepista personata*, *P. gilva* and/or *Tricholoma equestre* fruit bodies belonging to the Tricholomataceae family suggests that such species may be characterised by the most effective accumulation of selected major or trace elements. On the other hand, mushrooms belonging to the Agaricaceae family (*Agaricus arvensis*, *Coprinus comatus* and *Macrolepiota procera*) were characterised by significant differences in the content of all determined elements jointly, which suggests that a higher content of one or several elements is mushroom species-dependent.

**Figure:**
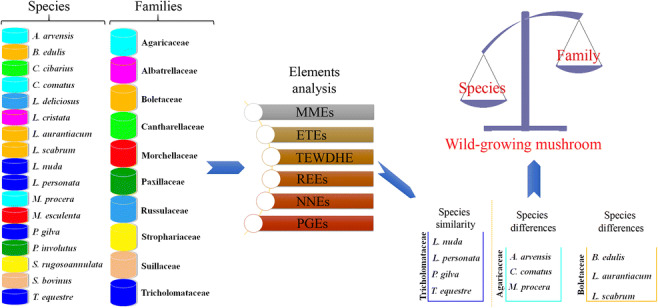
Graphical abstract

Graphical abstract

## Introduction

The accumulation of elements by wild-growing mushrooms has been the subject of numerous scientific papers around the world (e.g. Campos and Tejera 2011; Falandysz and Drewnowska 2015; Rudawska and Leski 2005; Sun et al. 2017). Depending on the site of the fruit body collection, a higher, lower or significantly differentiated ability of mushrooms to accumulate individual elements or some elements was reported (Braeuer et al. 2018; Melgar et al. 2016). Kalač (2010, 2019) has described ranges in the content of individual elements based on available literature data. This made it possible to indicate species with higher but also limited ability to collect elements from underlying soil (Kalač and Svoboda 2000). Many of the studied mushroom species have a wide range in the content of elements in their fruit bodies, e.g. Ca in *Boletus edulis* or *Cantharellus cibarius*, K in *Lactarius deliciosus*, P in *Tremella fuciformis*, Cu in *Macrolepiota procera* or Zn in *Boletus edulis*. It should be emphasised that, in most cases, the diversified efficiency of accumulation of certain elements resulted from differences in their concentration in the soil. Examples can be found in the studies of Radulescu et al. (2010) and Zavastin et al. (2018), in which *Armillaria mellea* growing on unpolluted and polluted substrates was associated with significantly different contents of Se. However, the efficiency of element accumulation does not always depend on their concentration in a substrate (Radulescu et al. 2010; Sarikurkcu et al. 2015). This suggests that the element contents in such cases depend on mushroom species, genus or the families to which they belong.

Mushroom families with high and low levels of accumulation of individual major elements have been investigated by Seeger (1978), Seeger and Beckert (1979), Seeger and Hüttner (1981) and Seeger et al. (1983) for K, Mg, Ca and Na, respectively. On the other hand, a review of Falandysz and Borovička (2013) as well as an original paper by Sácký et al. (2016) indicates that the efficiency of selected element(s) accumulation by individual mushroom species (mainly for hyperaccumulation) depends on their genetic properties. To demonstrate the higher efficiency of element accumulation by species belonging to a particular family, it is necessary to collect the fruit bodies of many other species from the same and different sites. It is important to show that, regardless of the substrate composition, the fruit bodies included in this family contain a higher/lower amount of elements (Kalač 2019). Unfortunately, while such a comparison in the case of cultivated mushrooms is relatively simple to make, it is challenging for wild-growing mushroom species due to the impact of environmental factors and the growth of fruit bodies on different soils and at different times. Only the collection of a great number of fruit bodies of different species, or genus belonging to several families from a large number of locations, can indicate which taxonomic unit determines the mineral composition of mushrooms.

The aim of the study was to determine the content of 63 mineral elements in 17 wild-growing mushroom species and to elucidate similarities and differences in their ability to accumulate some of the elements. Fruit bodies were collected in Wielkopolska Province in Poland from sites significantly different as regards their substrate composition. The ability of the studied mushroom species to accumulate all the determined elements and 6 separate groups of elements was determined. The obtained values were used for an assessment of the so far unresolved question: is an ability to accumulate certain element(s) an attribute of a species or family?

## Materials and methods

### Experimental material

In these studies, 17 wild-growing mushroom species were analysed with respect to the content of 63 elements. Because 2018 was a very specific year in terms of the considerable quantities of mushrooms collected in Polish forests, fruit bodies were taken from up to 143 sites located in Wielkopolska Province between 7 September and 29 October 2018. Sampling sites were limited to only half of the area of one administrative unit of Poland with the intent to minimise the variability of climatic, soil and geographical conditions. Fruit bodies of particular mushroom species were collected from different places such as pine, beech, deciduous or mixed forests, gardens, mulches, city parks or meadows, intentionally to verify the hypothesis that mushroom species belonging to the same family show higher or lower accumulation of selected groups of elements (Table 1).

**Table 1:** Characteristics of studied aboveground mushroom species

No.	Aboveground species	Family	Edibility	Nutritional strategy	Site	Amount of sample collection sites	Number of fruit bodies forming a sample
1.	*Agaricus arvensis* Schaeff.	Agaricaceae	Edible	Saprobic	City park	9	3
2.	*Boletus edulis* Bull.	Boletaceae	Edible	Mycorrhizal	Pine forest	27	2
3.	*Cantharellus cibarius* Fr.	Cantharellaceae	Edible	Mycorrhizal	Pine forest	18	4
4.	*Coprinus comatus* (O.F.Müll.) Pers.	Agaricaceae	Edible	Saprobic	Meadow	6	2
5.	*Lactarius deliciosus* (L.) Gray	Russulaceae	Edible	Mycorrhizal	Pine forest	22	3
6.	*Laeticutis cristata* (Schaeff.) Audet	Albatrellaceae	Non-edible	Saprobic	Deciduous forest	4	3
7.	*Leccinum aurantiacum* (Bull.) Gray	Boletaceae	Edible	Mycorrhizal	Deciduous forest	15	4
8.	*Leccinum scabrum* (Bull.) Gray	Boletaceae	Edible	Mycorrhizal	Deciduous forest	31	3
9.	*Lepista nuda* (Bull.) Cooke	Tricholomataceae	Edible	Saprobic	Beech forest	17	3
10.	*Lepista personata* (Fr.) Cooke	Tricholomataceae	Edible	Saprobic	Meadow	7	2
11.	*Macrolepiota procera* (Scop.) Singer	Agaricaceae	Edible	Saprobic	Meadow near the forest	8	2
12.	*Morchella esculenta* (L.) Pers.	Morchellaceae	Edible	Saprobic/mycorrhizal	Garden, mulch	11	3
13.	*Paralepista gilva* (Pers.) Raithelh	Tricholomataceae	Edible	Saprobic	Mixed forest	22	5
14.	*Paxillus involutus* (Batsch) Fr.	Paxillaceae	Non-edible	Saprobic	City park	6	3
15.	*Stropharia rugosoannulata* Farl. ex Murrill	Strophariaceae	Edible, young fruiting bodies	Saprobic	Garden, mulch	14	4
16.	*Suillus bovinus* (L.) Roussel	Suillaceae	Edible	Mycorrhizal	Young pine forest	9	3
17.	*Tricholoma equestre* (L.) P.Kumm.	Tricholomataceae	Edible(?)	Mycorrhizal	Pine forest	30	3

Some fruit bodies of different mushroom species were collected from the same sites

The 17 studied mushroom species belong to 10 families: Agaricaceae (3), Albatrellaceae (1), Boletaceae (3), Cantharellaceae (1), Morchellaceae (1), Paxillaceae (1), Russulaceae (1), Strophariaceae (1), Suillaceae (1) and Tricholomataceae (4). The fresh weight of the complete fruit bodies collected from each site and mushroom species was at least 32.3 g.

### Analytical procedure

Collected fruit bodies were washed with deionised ultrapure water (Milli-Q, Millipore, Saint Luis, USA) to remove the remaining soil particles and possible element ions adsorbed on their surface. Samples were preliminarily dried at 65 ± 1 °C for 120 h in an electric oven (SLW 53 STD, Pol-Eko, Wodzisław Śląski, Poland) and ground in a laboratory mill SM 200 (Retsch GmbH, Haan, Germany) to obtain a powder. 0.300 ± 0.001 g of a sample was digested by 10 mL of concentrated nitric acid (65%; Sigma-Aldrich, St. Louis, MO, USA) in closed Teflon containers in the microwave sample preparation system Mars 6 Xpress. After digestion, cold samples were filtered (Qualitative Filter Papers Whatman) and diluted by water to a final volume of 15.0 mL. The inductively coupled plasma spectrometer with optical emission detection (Agilent 5110 ICP-OES, Agilent USA) was used for sample analysis. In all determinations, common conditions were used: plasma gas flow 12.0 L min^−1^, nebuliser gas flow 0.7 L min^−1^, auxiliary gas flow 1.0 L min^−1^, radio frequency (RF) power 1.2 kW. The most sensitive analytical wavelengths were used for all element determinations, additionally, for selected elements (Al, Ca, Fe, K, Mg, Na and P), and alternative less sensitive wavelengths were used to increase the range of calibration. Commercial ICP analytical standards (Romil, England) and demineralised water (Direct-Q system, Millipore, USA) were used for the calibration. The detection limits were estimated in the range of 0.01–0.09 mg kg^−1^ dry weight (DW) using the criteria of 3-sigma. The uncertainty level was estimated for the procedure, including sample preparation at the level of 20%. Both certified reference materials analysis (soils: CRM and CRM S-1; sediments: CRM 667 and CRM 405; CRM NCSDC (73349)—bush branches and leaves) and the standard addition method was used in quality control with acceptable recovery (80–120%). All the determined elements were divided into 6 groups of elements, according to Kalač (2019):

i. Major essential elements (MEEs): Ca, Mg, K, Na and P;
ii. Essential trace elements (ETEs): B, Co, Cu, Cr, Fe, Mn, Mo, Ni, Se and Zn;
iii. Trace elements with detrimental health effect (TEWDHE): Ag, As, Ba, Be, Cd, Hg, Pb and Tl;
iv. Rare earth elements (REEs): Ce, Dy, Er, Eu, Gd, Ho, La, Lu, Nd, Pr, Sc, Sm, Tb, Tm, Y and Yb;
v. Nutritionally non-essential elements (NNEs): Al, Au, Bi, Ga, Ge, Hf, In, Li, Rb, Re, Sb, Sc, Sr, Te, Th, Ti, U, V and Zr;
vi. Platinum group elements (PGEs): Ir, Os, Pd, Pt, Rh, Ru.

### Statistical analysis

To compare the mean content of 63 elements in 17 wild-growing mushroom species, the one-way ANOVA and also Tukey’s HSD (statistically significant difference) test were used. Results below the limit of detection were applied as the half of the detection limit values.

Because all determined elements were divided into 6 separate groups of elements, for each of them separately but also for all 63 elements jointly, a principal component analysis (PCA) was performed as an initial graphical presentation of the obtained results (Morrison 1990; Abdi and Williams 2010) to show the relationship between all the studied mushroom species. Additionally, for better visualization of multidimensional data (mean concentration of particular 63 elements in studied mushrooms), a Heatmap with a cluster analysis was performed. To show similarities between particular mushroom species as regards the content of elements belonging to particular groups of elements, cluster analysis for six groups of elements was conducted using the Ward method, and particular cases were grouped based on Euclidean distance. The same analysis was used to compare the content of 6 particular groups of elements and all elements jointly determined in all 17 mushroom species. Finally, the rank sum was performed to explain which mushroom species was the most enriched with all determined elements. A supplement for this analysis was the calculation of the Spearman rank correlation coefficients (*r*_s_) between particular groups of elements, being a nonparametric measure of rank correlation. All the statistical analyses were performed using the agricole package (R) and STATISTICA 12.0 software (StatSoft, USA).

## Results

### Content of elements in mushroom species

All data on mineral element contents are expressed in mg kg^−1^ dry weight.

Content of MEEs in the studied group of mushroom species significantly differed (Table 2). Ranges of mean contents of Ca, Mg, K, Na and P were from 34.0 to 531 (*S. rugosoannulata* and *L. nuda*), 265 to 1060 (*L. scabrum* and *L. personata*), 5780 to 41400 (*L. cristata* and *C. comatus*), 40.0 to 463 (*L. cristata* and *B. edulis*) and 1850 to 15800 mg kg^−1^ (*T. equestre* and *A. arvensis*), respectively. It is worth underlining that *C. comatus* was characterised by the highest content (next to *L. personata*) of Mg (973 mg kg^−1^) and also a high content of Na and P (286 and 12800 mg kg^−1^, respectively).

**Table 2:** Content of major essential elements (mg kg^−1^) in studied aboveground mushroom species

Mushroom species	Ca	Mg	K	Na	P
*A. arvensis*	340^c^	827^ab^	19000^ef^	43.1^f^	15800^a^
*B. edulis*	73.7^fg^	380^cd^	19000^ef^	463^a^	6120^efg^
*C. cibarius*	47.2^g^	572^bcd^	39600^ab^	181^cd^	4690^fg^
*C. comatus*	57.5^fg^	973^a^	41400^a^	286^b^	12800^abc^
*L. deliciosus*	172^d^	521^bcd^	16000^efg^	65.4^f^	5760^efg^
*L. cristata*	37.9^g^	389^cd^	5780^g^	40.0^f^	7580^ef^
*L. aurantiacum*	66.6^fg^	689^abc^	35400^abc^	78.6^ef^	9610b^cde^
*L. scabrum*	133^de^	265^d^	17400^ef^	277^bc^	5920^efg^
*L. nuda*	531^a^	836^ab^	33500^a–d^	96.6^def^	13700^b^
*L. personata*	97.1^ef^	1060^a^	26100^cde^	172^de^	12600^a–d^
*M. procera*	52.6^g^	816^ab^	31700^a–d^	288^b^	8210^def^
*M. esculenta*	416^b^	704^abc^	14400^fg^	74.4^ef^	9410^b–e^
*P. gilva*	163^d^	881^ab^	34400^abc^	87.5^def^	14800^a^
*P. involutus*	74.7^fg^	518^bcd^	33800^a–d^	64.0^f^	8320^c–f^
*S. rugosoannulata*	34.0^g^	555^bcd^	30400^bcd^	55.6^f^	6910^ef^
*S. bovinus*	147^d^	316^cd^	15000^fg^	66.3^f^	6280^efg^
*T. equestre*	65.8^fg^	405^cd^	23500^def^	50.6^f^	1850^g^

Identical superscripts denote non-significant differences between means within columns according to the post hoc Tukey’s HSD test

Significant differences between ETEs were also observed (Table 3), as reflected in the wide ranges between extreme contents of particular elements between selected mushroom species. These ranged from 0.422 and 0.423 to 71.9 (*A. arvensis*, *L. scabrum* and *P. involutus*, respectively), 4.17 to 204 (*L. cristata* and *M. procera*), 35.9 to 425 (*L. cristata* and *L. nuda*), 8.60 to 115 (*P. involutus* and *B. edulis*), 0.105 to 1.72 (*S. bovinus* and *L. personata*), 0.148 to 1.20 (*S. rugosoannulata* and *M. esculenta*), 0.130 to 8.55 (*M. esculenta* and *A. arvensis*) and 17.2 to 252 mg kg^−1^ (*L. cristata* and *A. arvensis*) for B, Cu, Fe, Mn, Mo, Ni, Se and Zn, respectively. *Cantharellus cibarius* was the only one of the studied mushroom species found to contain Co above the limit of detection (0.070 mg kg^−1^); similarly, only *M. esculenta* and *T. equestre* contained detectable levels of Cr (1.76 and 0.348 mg kg^−1^, respectively).

**Table 3:** Content of essential trace elements (mg kg^−1^) in studied aboveground mushroom species

Mushroom species	B	Co	Cu	Cr	Fe	Mn	Mo	Ni	Se	Zn
*A. arvensis*	0.423^c^	bDL	23.8^d–g^	bDL	103^cdef^	20.4^fg^	0.145^d^	0.247^c^	8.55^a^	252^a^
*B. edulis*	bDL	bDL	22.6^d–g^	bDL	57.1^ef^	115^a^	0.106^d^	0.786^abc^	6.29^b^	185^ab^
*C. cibarius*	4.03^bc^	0.070	34.8^def^	bDL	142^b–e^	62.2^cd^	bDL	0.270^bc^	0.431^d^	108^c–h^
*C. comatus*	bDL	bDL	37.4^de^	bDL	96.4^c–f^	10.8^g^	bDL	0.539^abc^	bDL	86.4^e–i^
*L. deliciosus*	3.48^bc^	bDL	10.9^efg^	bDL	85.5^c–f^	18.1^fg^	bDL	0.634^abc^	bDL	124^b–g^
*L. cristata*	bDL	bDL	4.17^g^	bDL	35.9^f^	12.8^g^	0.291^cd^	0.153^c^	1.46^cd^	17.2^i^
*L. aurantiacum*	1.05^c^	bDL	10.7^fg^	bDL	83.9^c–f^	13.7^fg^	0.109^d^	0.280^bc^	0.514^cd^	102^d–h^
*L. scabrum*	0.423^c^	bDL	6.2^g^	bDL	66.2^ef^	16.4^fg^	0.122^d^	0.967^ab^	0.550^cd^	54.2^ghi^
*L. nuda*	18.1^b^	bDL	86.8^c^	bDL	425^a^	49.9^de^	1.13^b^	0.482^bc^	1.07^cd^	140^b–e^
*L. personata*	14.6^bc^	bDL	135^b^	bDL	179^bcd^	15.4^fg^	1.72^a^	0.489^abc^	0.374^d^	180^abc^
*M. procera*	9.77^bc^	bDL	204^a^	bDL	77.5^def^	96.8^ab^	0.336^cd^	0.363^bc^	1.85^c^	135^b–f^
*M. esculenta*	5.87^bc^	bDL	19.2^efg^	1.76^a^	237^b^	44.4^de^	0.367^cd^	1.20^a^	0.130^d^	173^bcd^
*P. gilva*	66.3^a^	bDL	45.9^d^	bDL	105^c–f^	74.9^bc^	0.519^c^	0.624^abc^	5.45^b^	155^b–e^
*P. involutus*	71.9^a^	bDL	35.4^def^	bDL	125^c–f^	8.60^g^	bDL	0.256^bc^	0.398^d^	179^abc^
*S. rugosoannulata*	bDL	bDL	13.6^efg^	bDL	185^bc^	35.9^ef^	bDL	0.148^c^	bDL	62.3^f–i^
*S. bovinus*	5.81^bc^	bDL	4.38^g^	bDL	61.5^ef^	14.5^fg^	0.105^d^	0.184^c^	0.674^cd^	43.5^hi^
*T. equestre*	2.34^bc^	bDL	5.43^g^	0.348^b^	379^a^	13.2^g^	bDL	0.381^bc^	0.611^c^	63.7^f–i^

*bDL* value below detection limit; identical superscripts denote non-significant differences between means within columns according to the post hoc Tukey’s HSD test

Considering food security, a special group of elements are toxic trace elements with detrimental health effects (TEWDHE) (Table 4). In this group, the ranges for Ag, As, Ba, Cd, Hg, Pb and Tl were from 0.111 to 7.00 (*M. esculenta* and *A. arvensis*), 0.107 to 2.21 (*P. involutus* and *S. bovinus*), 0.014 to 7.29 (*L. cristata* and *P. gilva*), 0.175 and 3.49 (*L. cristata* and *L. deliciosus*), 0.246 to 4.23 (*S. bovinus* and *M. procera*), 0.524 to 7.54 (*S. bovinus* and *L. deliciosus*) and 0.189 to 2.89 mg kg^−1^ (*L. scabrum* and *L. cristata*), respectively. *Lepista nuda*, *M. procera* and *M. esculenta* were the only three mushroom species with a content of Be above the limit of detection (0.063; 0.094 and 0.013 mg kg^−1^, respectively).

**Table 4:** Content of trace elements with detrimental health effect (mg kg^−1^) in studied aboveground mushroom species

Mushroom species	Ag	As	Ba	Be	Cd	Hg	Pb	Tl
*A. arvensis*	7.00^a^	1.43^bc^	3.03^b–f^	bDL	1.19^bde^	0.421^gh^	1.76^cde^	bDL
*B. edulis*	1.62^cd^	0.130^fg^	2.39^b–f^	bDL	2.47^ab^	0.976^bcd^	1.95^cde^	0.237^d^
*C. cibarius*	0.361^e^	1.34^cd^	5.45^abc^	bDL	0.713^e^	0.697^ef^	1.93^cde^	0.951^bc^
*C. comatus*	0.175^e^	0.277^fg^	1.18^ef^	bDL	0.669^e^	1.01^bc^	0.930^de^	bDL
*L. deliciosus*	2.38^c^	0.741^c–g^	4.18^a–e^	bDL	3.49^a^	0.706^e^	7.54^a^	bDL
*L. cristata*	0.144^e^	2.17^ab^	0.014^f^	bDL	0.175^e^	0.420^gh^	2.57^b–e^	2.89^a^
*L. aurantiacum*	0.208^e^	0.402^efg^	2.86^b–f^	bDL	2.15^bd^	0.434^fgh^	1.56^cde^	bDL
*L. scabrum*	0.155^e^	0.140^fg^	2.33^c–f^	bDL	0.872^de^	0.270^h^	3.94^bc^	0.189^d^
*L. nuda*	0.821^de^	0.605d^efg^	4.27^a–e^	0.063^b^	0.499^e^	0.645^efg^	2.97^bcd^	1.39^b^
*L. personata*	1.07^de^	0.118^fg^	2.66^b–f^	bDL	0.403^e^	0.394^gh^	3.44^bc^	1.44^b^
*M. procera*	0.612^de^	0.881^c–f^	1.95^def^	0.094^a^	0.931^de^	4.23^a^	3.54^bc^	bDL
*M. esculenta*	0.111^e^	1.06^cde^	6.56^a^	0.013^c^	0.943^de^	0.703^e^	3.15^bcd^	bDL
*P. gilva*	5.56^be^	0.165^fg^	7.29^a^	bDL	1.37^bde^	0.766^cde^	4.42^b^	bDL
*P. involutus*	0.963^de^	0.107^g^	5.70^ab^	bDL	0.578^e^	1.20^b^	1.77^cde^	0.651^cd^
*S. rugosoannulata*	0.142^e^	0.300^efg^	1.03^ef^	bDL	0.367^e^	0.580^efg^	2.01^b–e^	1.54^b^
*S. bovinus*	0.340^e^	2.21^a^	2.55^b–f^	bDL	0.825^e^	0.246^h^	0.524^e^	1.41^b^
*T. equestre*	0.609^de^	2.13^ab^	4.54^a–d^	bDL	0.672^e^	0.740^de^	2.44^b–e^	0.529^cd^

*bDL* value below detection limit; identical superscripts denote non-significant differences between means within columns according to the post hoc Tukey’s HSD test

The greater proportion of the content of rare earth elements (REEs) was below the detection limits (Table 5). The highest sum of mean content of individual REEs was found in *L. cristata* and *M. esculenta* (4.95 and 4.47 mg kg^−1^, respectively), while the lowest in *B. edulis* fruit bodies (0.658 mg kg^−1^). The mean content for all analysed mushrooms was 2.40 mg kg^−1^. *L. cristata* was the species with the highest contents of Dy (together with *S. bovinus*), Er, Ho, Nd, Sm, Tb and Yb (0.196; 0.288; 0.634; 2.04; 0.063; 0.333 and 0.030 mg kg^−1^, respectively), while *M. esculenta* contained the highest level of Ce, Lu and Y (0.758; 0.287 and 0.235 mg kg^−1^, respectively). The highest La content of 0.443 mg kg^−1^ was observed in *P. gilva*.

**Table 5:** Content of rare earth elements (mg kg^−1^) in studied aboveground mushroom species

Mushroom species	Ce	Dy	Er	Eu	Gd	Ho	La	Lu	Nd	Pr	Sm	Tb	Tm	Y	Yb	∑_REEs_
*A. arvensis*	bDL	0.051^b^	bDL	bDL	0.005^b^	0.053^d^	0.076^c^	bDL	0.594^bc^	0.190^de^	bDL	bDL	bDL	0.053^ab^	bDL	1.02^f^
*B. edulis*	bDL	bDL	bDL	bDL	bDL	0.165^d^	0.065^c^	bDL	0.270^c^	0.102^e^	bDL	bDL	0.013^b^	0.043^ab^	bDL	0.658^f^
*C. cibarius*	bDL	bDL	bDL	0.019^cd^	0.014^b^	bDL	0.152^bc^	0.016^b^	0.309^c^	1.78^abc^	0.114^a^	0.069^c^	0.093^b^	0.024^b^	bDL	2.59^b–f^
*C. comatus*	bDL	bDL	bDL	0.100^ab^	0.050^b^	bDL	0.063^c^	0.043^b^	0.473^bc^	0.845^cde^	bDL	0.006^d^	0.048^b^	0.057^ab^	bDL	1.68^def^
*L. deliciosus*	bDL	0.007^b^	bDL	0.068^a–d^	bDL	0.068^d^	0.050^c^	bDL	0.303^c^	1.91^ab^	bDL	bDL	bDL	0.052^ab^	bDL	2.46^c–f^
*L. cristata*	bDL	0.196^a^	0.288^a^	bDL	0.150^ab^	0.634^a^	0.087^bc^	0.037^b^	2.04^a^	0.785^cde^	0.063^b^	0.333^a^	0.252^ab^	0.050^ab^	0.030^a^	4.95^a^
*L. aurantiacum*	0.052^b^	0.023^b^	0.103^a^	0.132^a^	0.085^ab^	0.063^d^	0.042^c^	0.024^b^	0.375^c^	1.39^abc^	bDL	0.044^cd^	0.018^b^	0.047a^b^	0.011^ab^	2.41^c–f^
*L. scabrum*	bDL	bDL	bDL	0.009^d^	0.032^b^	0.095^d^	0.052^c^	bDL	0.364^c^	1.22^bcd^	bDL	bDL	0.043^b^	0.050^ab^	bDL	1.87^def^
*L. nuda*	bDL	bDL	0.019^a^	bDL	bDL	0.101^d^	0.009^c^	bDL	0.654^bc^	0.146^e^	bDL	0.059^c^	0.084^b^	0.033^b^	0.005^b^	1.11^f^
*L. personata*	bDL	0.009^b^	bDL	0.052^bcd^	bDL	bDL	0.156b^c^	0.099^b^	0.387^bc^	0.767^cde^	bDL	0.007^d^	bDL	0.037^b^	bDL	1.51^def^
*M. procera*	0.039^b^	0.023^b^	0.043^a^	0.046^bcd^	0.312^a^	0.064^d^	0.020^c^	0.034^b^	0.458^bc^	2.36^a^	bDL	bDL	0.449^a^	0.027^b^	0.014^ab^	3.89^abc^
*M. esculenta*	0.758^a^	0.104^ab^	0.098^a^	0.071^a–d^	0.111^ab^	0.119^d^	0.424^a^	0.287^a^	0.831^b^	1.31^bc^	bDL	0.029^cd^	0.080^b^	0.235^a^	0.022^ab^	4.47^ab^
*P. gilva*	0.055^b^	0.044^b^	0.013^a^	0.057^bcd^	0.105^ab^	0.124^d^	0.443^a^	bDL	0.638^bc^	1.38^abc^	bDL	bDL	0.149^b^	0.128^ab^	0.009^ab^	3.15^a–e^
*P. involutus*	0.044^b^	0.031^b^	bDL	0.042^bcd^	bDL	0.313^b^	0.135^bc^	0.025^b^	0.586^bc^	1.06^bcde^	bDL	bDL	bDL	0.061^ab^	0.016^ab^	2.32^c–f^
*S. rugosoannulata*	bDL	0.022^b^	0.128^a^	bDL	0.029^b^	0.307^bc^	0.074^c^	0.004^b^	0.473^bc^	0.130^e^	bDL	bDL	0.006^b^	0.011^b^	bDL	1.18^ef^
*S. bovinus*	0.156^b^	0.031^b^	0.343^a^	0.085^abc^	0.069^b^	0.126^d^	0.060^c^	0.016^b^	0.391^bc^	0.763^cde^	bDL	0.125^b^	0.028^b^	0.020^b^	0.016^ab^	2.23^c–f^
*T. equestre*	0.604^ab^	0.103^ab^	0.062^a^	0.065^a–d^	0.064^b^	0.172^cd^	0.328^ab^	bDL	0.576^bc^	1.10^bcde^	bDL	0.050^cd^	0.082^b^	0.116^ab^	0.024^ab^	3.35^a–d^

*bDL* value below detection limit; identical superscripts denote non-significant differences between means within columns according to the post hoc Tukey’s HSD test

The contents of several elements in some species were also below detection limits within the group of NNEs (Table 6). The ranges of mean contents (mg kg^−1^) were from 0.849 to 160 (*L. cristata* and *P. gilva*) for Al, 0.219 to 2.64 (*M. procera* and *L. cristata*) for Bi, 0.028 to 0.456 (*L. personata* and *M. esculenta*) for Ga, 0.070 to 2.58 (*L. personata* and *L. cristata*) for Ge, 1.13 to 7.60 (*L. personata* and *L. cristata*) for In, 0.015 to 0.709 (*L. personata* and *M. esculenta*) for Li, 1.22 to 389 (*M. procera* and *C. cibarius*) for Rb, 0.011 to 0.630 (*M. procera* and *L. cristata*) for Re, 0.007 to 0.064 (*L. deliciosus* and *M. esculenta*) for Sc, 0.581 to 9.16 (*L. cristata* and *M. esculenta*) for Sr, 0.315 to 5.37 (*C. comatus* and *A. arvensis*) for Te, 0.010 to 2.09 (*L. cristata* and *P. gilva*) for Ti, 0.010 to 1.05 (*L. scabrum* and *M. esculenta*) for U, 0.010 to 0.624 (*B. edulis* and *P. gilva*) for V and 0.008 to 0.087 (*L. deliciosus* and *P. involutus*) for Zr. Four elements within the NTEs were detectable singularly. Content of Au above the limit of detection was only determined in *L. personata* fruit bodies (0.026 mg kg^−1^), similarly for Hf in *S. bovinus* (0.080 mg kg^−1^), Sb in *C. comatus* and *L. cristata* (0.698 and 0.406 mg kg^−1^, respectively) and Th in *S. rugosoannulata* (0.092 mg kg^−1^).

**Table 6:** Content of nutritionally trace elements (mg kg^−1^) in studied aboveground mushroom species

Mushroom species	Al	Au	Bi	Ga	Ge	Hf	In	Li	Rb	Re	Sb	Sc	Sr	Te	Th	Ti	U	V	Zr
*A. arvensis*	29.1^cd^	bDL	1.64b^c^	0.272^bcd^	0.356^cde^	bDL	5.04^a–d^	bDL	bDL	0.154^cd^	bDL	bDL	1.07^b^	5.37^a^	bDL	0.117^c^	0.369^bcd^	0.022^c^	0.035^abc^
*B. edulis*	10.8^de^	bDL	0.969^c^	0.073^ef^	0.321^cde^	bDL	6.55^ab^	bDL	100^bc^	bDL	bDL	0.014^b^	1.44^b^	1.79^def^	bDL	0.359^bc^	0.098^cd^	0.007^c^	0.031^abc^
*C. cibarius*	99.3^b^	bDL	bDL	bDL	0.806^bcd^	bDL	4.69^a–e^	0.026^c^	389^a^	bDL	bDL	0.020^b^	3.74^ab^	2.00^def^	bDL	0.864^abc^	0.246^bcd^	0.068^c^	0.046^abc^
*C. comatus*	24.5^cde^	bDL	bDL	bDL	bDL	bDL	3.13^b–e^	bDL	bDL	bDL	0.698^a^	bDL	1.26^b^	0.32^f^	bDL	0.152^c^	0.028^cd^	0.023^c^	0.029^abc^
*L. deliciosus*	23.1^cde^	bDL	2.03^ab^	0.035^f^	0.337^cde^	bDL	3.24^b–e^	bDL	135^b^	0.021^ef^	bDL	0.006^b^	2.15^b^	2.74^cde^	bDL	0.206^bc^	0.274^bcd^	0.016^c^	0.007^c^
*L. cristata*	0.849^e^	bDL	2.64^a^	0.379^ab^	2.58^a^	bDL	7.60^a^	0.468^ab^	bDL	0.630^a^	0.406^b^	0.008^b^	0.58^b^	0.86^ef^	bDL	0.010^c^	0.734^ab^	0.057^c^	0.010^bc^
*L. aurantiacum*	14.6^de^	bDL	1.21^c^	0.243^bcd^	0.829^bcd^	bDL	3.67^b–e^	bDL	59.8^cde^	0.112^c–f^	bDL	0.008^b^	2.19^ab^	4.29^abc^	bDL	0.264^bc^	0.197^bcd^	0.044^c^	0.050^abc^
*L. scabrum*	14.0^de^	bDL	bDL	0.143^def^	bDL	bDL	5.52^abc^	bDL	14.0^de^	0.216^cb^	bDL	0.024^b^	1.22^b^	4.96^ab^	bDL	0.104^c^	0.000^d^	0.030^c^	0.043^abc^
*L. nuda*	23.7^cde^	bDL	1.12^c^	0.079^ef^	0.833^bcd^	bDL	1.54^de^	bDL	9.35^de^	0.080^def^	bDL	0.010^b^	1.21^b^	0.53^f^	bDL	0.193^bc^	0.127^cd^	0.071^c^	0.020^abc^
*L. personata*	45.9^c^	0.026	1.08^c^	0.028^f^	0.070^e^	bDL	1.13^e^	0.015^c^	7.59^de^	bDL	bDL	0.009^b^	2.86^ab^	0.38^f^	bDL	0.461b^c^	0.102^cd^	0.158^bc^	0.038^abc^
*M. procera*	14.7^de^	bDL	0.219^d^	0.207^cde^	0.506^b–e^	bDL	4.58^a–e^	bDL	1.22^e^	0.011^f^	bDL	0.020^b^	1.90^b^	0.77^ef^	bDL	0.079^c^	0.598^abc^	0.093^c^	0.032^abc^
*M. esculenta*	83.2^b^	bDL	1.65^bc^	0.456^a^	1.09b	bDL	5.28^abc^	0.709^a^	bDL	0.140^cd^	bDL	0.064^a^	9.16^a^	0.80^ef^	bDL	1.18^abc^	1.05^a^	0.366^b^	0.062^abc^
*P. gilva*	160^a^	bDL	1.52^bc^	0.321^abc^	bDL	bDL	3.10^b–e^	0.244^bc^	53.2^cde^	bDL	bDL	0.028^ab^	4.54^ab^	0.73^ef^	bDL	2.09^a^	0.31^bcd^	0.624^a^	0.070^abc^
*P. involutus*	35.7^cd^	bDL	1.13^c^	0.207^cde^	bDL	bDL	5.95^abc^	0.036^c^	bDL	bDL	bDL	0.013^b^	3.12^ab^	3.01^bcd^	bDL	0.587^abc^	0.494^a–d^	0.060^c^	0.087^a^
*S. rugosoannulata*	15.7^de^	bDL	bDL	0.174^def^	0.128^de^	bDL	7.43^a^	bDL	7.61^de^	0.302^b^	bDL	bDL	0.75^b^	0.47^f^	0.092	0.167^c^	0.248^bcd^	0.036^c^	0.059^abc^
*S. bovinus*	20.6^cde^	bDL	1.06^c^	0.197^def^	0.477^b–e^	0.080	4.04^a–e^	0.112^bc^	67.0^cd^	0.120^cde^	bDL	0.009^b^	3.12^ab^	0.93^ef^	bDL	0.267^bc^	0.506^a–d^	0.020^c^	0.068^abc^
*T. equestre*	42.7^c^	bDL	2.11^a^	0.130^def^	0.955^bc^	bDL	2.35^cde^	0.089^bc^	99.3^bc^	0.139^dc^	bDL	0.037^a^	5.71^ab^	0.74^ef^	bDL	1.71^ab^	0.37^bcd^	0.197^bc^	0.086^ab^

*bDL* value below detection limit; identical superscripts denote non-significant differences between means within columns according to the post hoc Tukey’s HSD test

It is probable that the elements belonging to PGE showed the smallest differentiation within all the analysed mushroom species (Table 7). Iridium and Pd in *C. comatus* (0.627 and 0.065 mg kg^−1^, respectively) and *L. cristata* (2.57 and 0.197 mg kg^−1^, respectively) were the only ones detectable. Similarly, *L. cristata* and *L. scabrum* were the only two species with an Ru content above the limit of detection (0.228 and 0.104 mg kg^−1^, respectively).

**Table 7:** Content of platinum group elements (mg kg^−1^) in studied aboveground mushroom species

Mushroom species	Ir	Os	Pd	Pt	Rh	Ru
*A. arvensis*	bDL	0.021^a^	bDL	5.72^cd^	0.345^bc^	bDL
*B. edulis*	bDL	0.105^a^	bDL	3.61^cde^	0.227^bc^	bDL
*C. cibarius*	bDL	0.140^a^	bDL	4.59^cde^	0.487^ab^	bDL
*C. comatus*	0.627^a^	bDL	0.065^b^	3.59^cde^	0.263^bc^	bDL
*L. deliciosus*	bDL	bDL	bDL	1.82^e^	0.108^c^	bDL
*L. cristata*	2.57^b^	0.290^a^	0.197^a^	2.90^de^	0.793^a^	0.228^a^
*L. aurantiacum*	bDL	0.088^a^	bDL	3.02^cde^	0.208^bc^	bDL
*L. scabrum*	bDL	bDL	bDL	3.45^cde^	0.177^bc^	0.104^b^
*L. nuda*	bDL	bDL	bDL	4.78^cde^	0.220^bc^	bDL
*L. personata*	bDL	bDL	bDL	5.31^cde^	0.266^bc^	bDL
*M. procera*	bDL	0.103^a^	bDL	3.17^cde^	0.213^bc^	bDL
*M. esculenta*	bDL	0.135^a^	bDL	12.25^a^	0.222^bc^	bDL
*P. gilva*	bDL	0.101^a^	bDL	10.70^ab^	0.284^bc^	bDL
*P. involutus*	bDL	0.150^a^	bDL	3.87^cde^	0.263^bc^	bDL
*S. rugosoannulata*	bDL	bDL	bDL	4.79^cde^	0.099^c^	bDL
*S. bovinus*	bDL	0.293^a^	bDL	4.21^cde^	0.277^bc^	bDL
*T. equestre*	bDL	0.102^a^	bDL	6.83^bc^	0.262^bc^	bDL

*bDL* value below detection limit; identical superscripts denote non-significant differences between means within columns according to the post hoc Tukey’s HSD test

### Similarities and differences between mushroom species

**Fig. 1:**
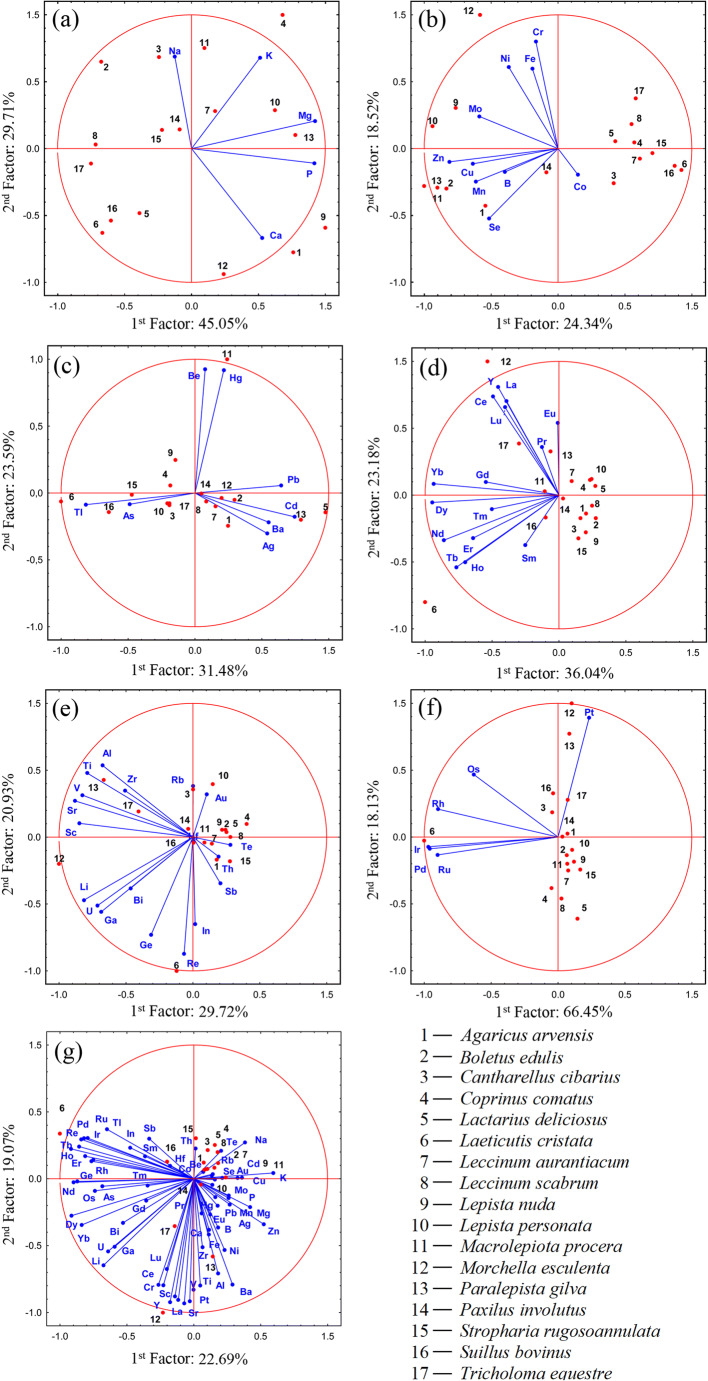
Principal component analysis for all studied wild-growing mushrooms concerning the content MEEs (a), ETEs (b), TEWDHE (c), REEs (d), NNEs (e), PGEs (f) and all elements jointly (g)

Relationships between the studied mushroom species within individual groups of elements were described using PCA (Fig. 1a–g).

In the case of MEEs, a clear ability for higher accumulation of K and Mg was observed in *C. comatus*, Mg and P in *P gilva* or Ca and P in *L. nuda* (Fig. 1a). On the other hand, the previously mentioned low content of K in *L. cristata* fruit bodies was also recorded. PCA for this group of elements explained 74.76% (45.05 + 29.71%) of total variability, which reliably reflects the relationships between the mushroom species.

Within ETEs, PCA explained only 42.86% (24.34 + 18.52), but this was enough to show that the studied mushroom species are generally divided into two groups with a higher or lower content of ETEs (Fig. 1b). Also, in this case, the previously mentioned highest contents of Ni were observed in *M. esculenta*, Mo in *L. personata*, Mn in *B. edulis* or Se in *A. arvensis*.

An interesting graphical distribution of mushroom species regarding the content of TEWDHE resulting from PCA is described in Fig. 1c, where 55.07% (31.48 + 23.59) of total variability was explained. In this case, 3 groups of mushrooms contained a higher content of (i) Be and Hg (*M. procera* or *S. bovinus*), (ii) As or Tl (e.g. *L. cristata*) and (iii) Ag, Ba, Cd and Pb (e.g. *A. arvensis*, *L. deliciosus* or *P. gilva*).

PCA for REEs explained 59.14% (36.04 + 23.18) of total variability, which showed that *L. cristata* and *M. esculenta* were the most metal enriched species in this group (Fig. 1d). Additionally, a separate group of mushroom species with low content of REEs was observed (e.g. *A. arvensis*, *B. edulis*, *L. deliciosus* or *L. nuda*).

Similar observations were recorded for NEEs, where PCA explained 50.65% (29.72 + 20.93) of the total variability (Fig. 1e). A distinctly higher total content of the NEEs was observed in *L. cristata*, *M. esculenta* and *P. gilva*.

In the case of PCA for PGEs, 84.58% (66.45 + 18.13) of total variability was explained. A higher content of Ir, Pd and also Rh and Ru was visible in *L. cristata*, similarly to Pt in *M. esculenta* and *P. gilva* (Fig. 1f). The other mushroom species created a separate group.

PCA calculated for all 63 elements jointly explained 41.76% (22.69 + 19.07) of total variability and has shown that some of the analysed mushroom species are able to accumulate one or more elements more effectively while accumulation in others is more limited (Fig 1g).

**Fig. 2:**
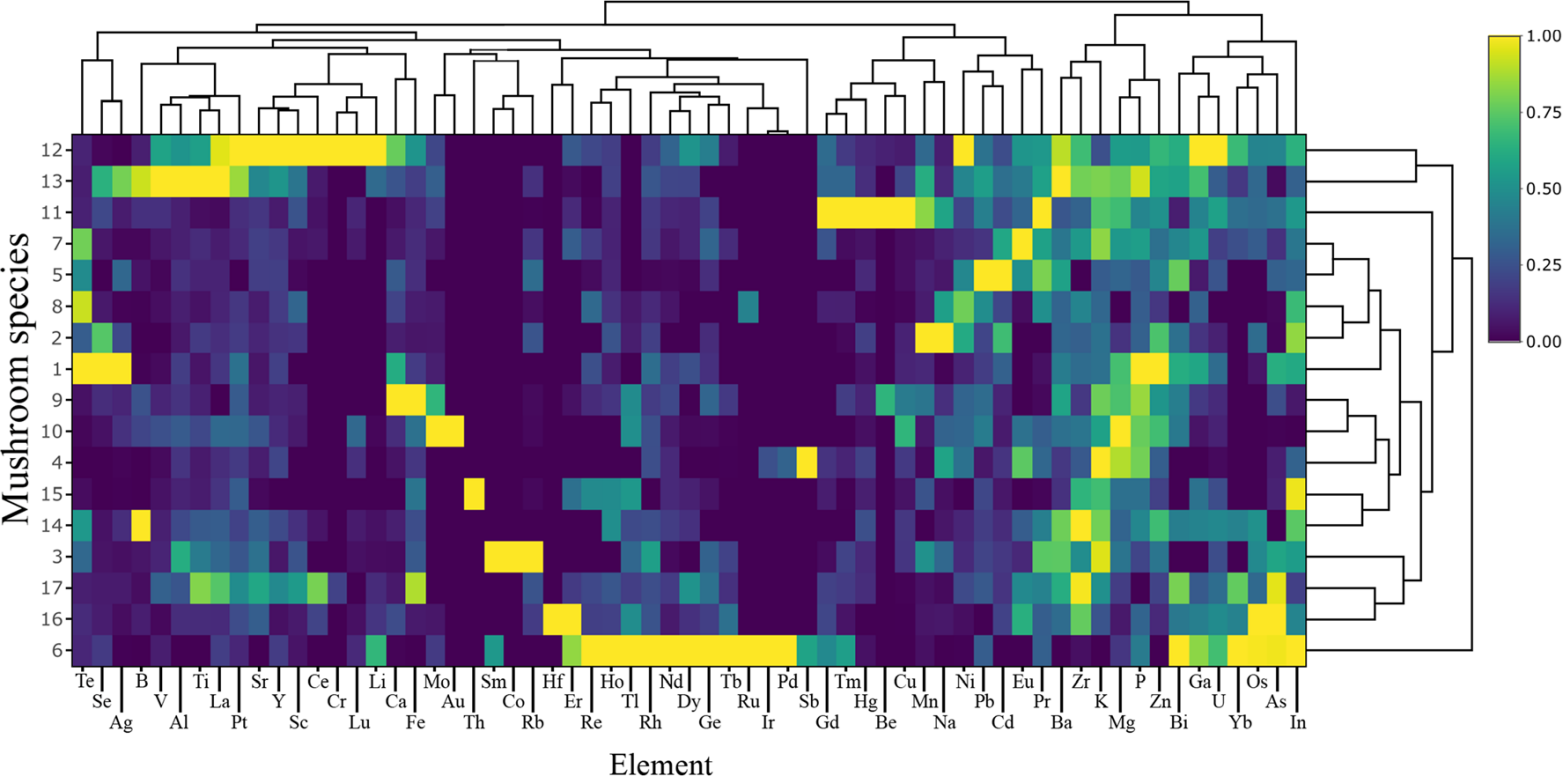
Correlations between 17 studied mushroom species concerning the content of all 63 elements (Heatmap) in mean values with presentation of a hierarchical tree plot

For this reason, a heatmap was prepared (Fig. 2) which allowed all 17 mushroom species to be compared with regard to the content of all 63 metal(loid)s. The highest similarities were recorded between *M. esculenta* and *P. gilva*, *P. involutus* and *S. rugosoannulata*, *S. bovinus* and *T. equestre*, *L. deliciosus* and *L. aurantiacum* or *B. edulis* and *L. scabrum.* The heatmap also showed which individual elements are accumulated similarly by all 17 mushroom species.

**Fig. 3:**
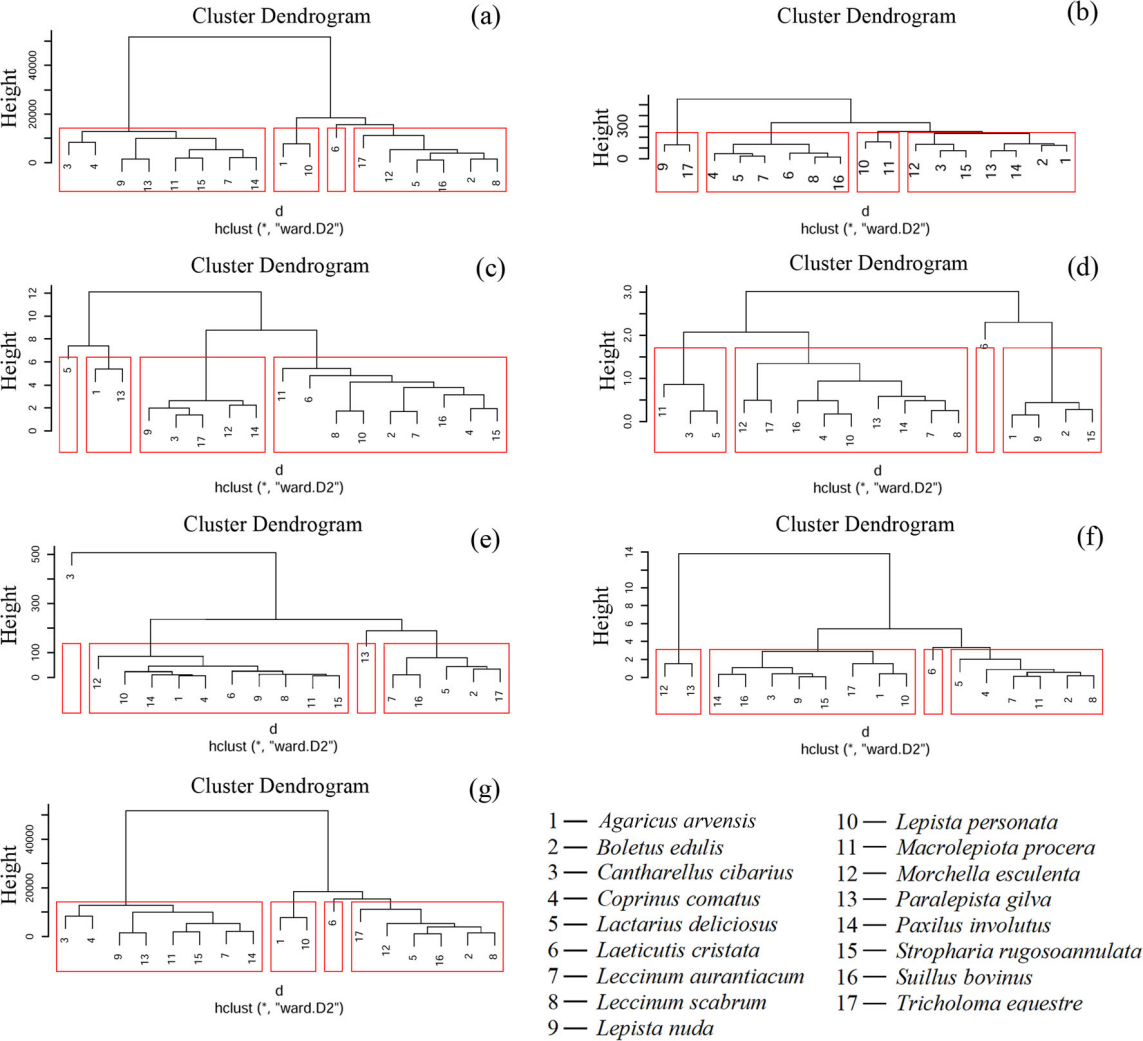
Cluster analysis to show the groups of similarly accumulated mushroom species of MEEs (a), ETEs (b), TEWDHE (c), REEs (d), NNEs (e), PGEs (f) and all elements jointly (g)

The obtained results led to the performance of a cluster analysis for six groups of elements using the Ward method (Fig. 3). For a particular group of elements, mushroom species were grouped based on Euclidean distance.

Within the MEE group, characterised by the lowest contents of Na and Ca, *L. cristata* formed a separate group, as did *A. arvensis* with *L. personata* (Fig 3a). The third group of species was composed of *B. edulis*, *L. scabrum*, *L. deliciosus*, *S. bovinus*, *M. esculenta* and *T. equestre*, while the last contained all the remaining mushroom species.

For ETEs, *L. personata* and *M. procera* constituted the first, while *L. nuda* and *T. equestre* the second group of mushroom species (Fig. 3b). *C. comatus*, *L. deliciosus*, *L. cristata*, *L. aurantiacum*, *L. scabrum* and *S. bovinus* formed the third of four groups.

Mushroom species were divided into four groups in accordance with the content of TEWDHE (Fig. 3c). The separate groups were composed of one (*L. deliciosus*), two (*A. arvensis* and *P. gilva*), five (*C. cibarius*, *L. nuda*, *M. esculenta*, *P. involutus* and *T. equestre*) and the remaining 9 mushroom species.

*Laeticutis cristata* was characterised by the highest contents of Dy, Er, Ho, Nd, Sm, Tb and Yb forming the first of the group within REEs, while *C. cibarius, L. deliciosus* and *M procera* formed the second (Fig. 3d). *A. arvensis*, *B. edulis*, *L. nuda* and S. *rugosoannulata* are included in the third group and the remaining 9 species constitute the fourth group.

As regards the content of NNEs, all 17 species were divided into four groups, composed of one (*C. cibarius* with the highest content of Rb), one (*P. gilva* containing the highest content of Al, Ti and V), five (*B. edulis*, *L. deliciosus*, *L. aurantiacum*, *S. rugosoannulata* and *T. euestre*) and the remaining 10 species (Fig. 3e).

Four groups were also appointed for mushroom species as regards the content of PGEs. *Leaticutis cristata* and *M. esculenta* with *P. gilva* forming the first and second groups, respectively, while *B. edulis*, *C. comatus*, *L. deliciosus*, *L. aurantiacum*, *L. scabrum* and *M. procera* belong to the third one (Fig. 3f). The rest of the 8 mushroom species constitute the fourth group.

Taking into consideration all 63 elements jointly, the 17 studied mushroom species were divided into 4 groups: the first (*L. cristata*), the second (*A. arvensis and L. personata*), the third (*B. edulis*, *L. deliciosus*, *L. scabrum*, *M. esculenta*, *S. bovinus* and *T. equestre*) and the fourth composed of the remaining 8 species (Fig. 3g).

**Fig. 4:**
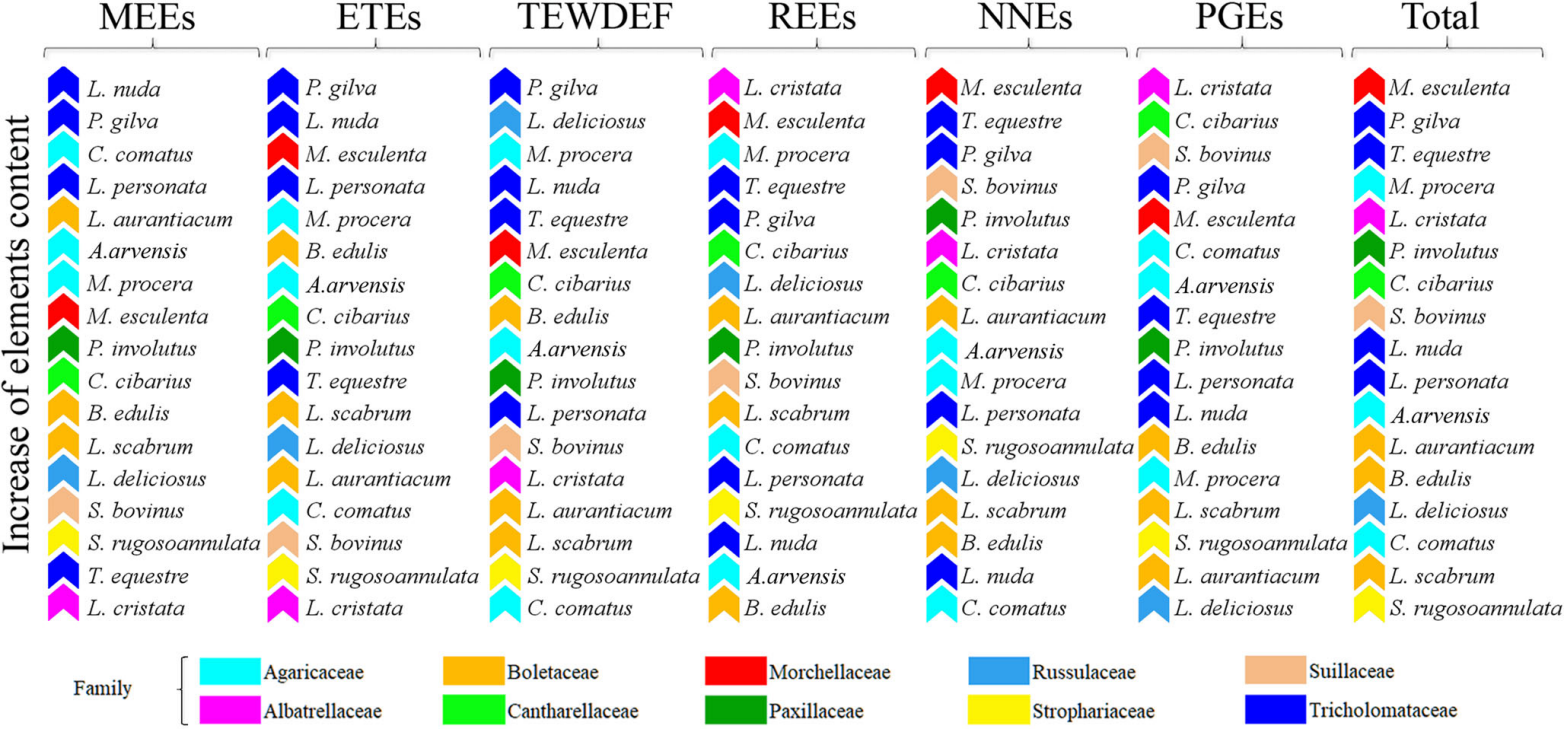
Graphical presentation of rank sum according to increase of the elements include to particular groups (MEEs, ETEs, TEWDHE, REEs, NNEs, PGEs) and all elements jointly in the studied mushroom species with their classification to individual families

The obtained results show extremely wide differences in the content of the studied elements, from several μg kg^−1^ of individual REEs to tens of g kg^−1^ of potassium. Unfortunately, they do not explain which one mushroom species was the most enriched with a particular group of elements or all elements jointly. A rank sum showed the highest content of MEEs and ETEs in *L. nuda* and *P. gilva* (Fig. 4), both species from the Tricholomataceae family. Inedible *Paralepista gilva* was also very rich in undesirable TEWDEF; however, it contained a relatively low content of REEs and NNEs. These groups of elements were dominant in *M. esculenta*, *T. equestre* or *L. cristata*. The latter species also contained the highest level of PGEs.

The data presented in Fig. 4 suggest that species belonging to the Tricholomataceae family were the most effective in MEEs, ETEs and TEWDEF accumulation, although with some exceptions. The opposite situation was observed for *B. edulis* and *L. scabrum*, characterised by a generally low content of the studied elements. Nevertheless, this was not found for *L. aurantiacum*, also from the Boletaceae family. Species of the Agaricaceae family were characterised by a relatively high content of MEEs and TEWDEF, while for the other four groups of elements, their different contents were species-dependent. The obtained results indicate that belonging of a given species to a particular family of mushrooms may determine a higher or lower content of a given group of elements. However, it is more likely that the efficiency of individual element accumulation depends primarily on the species.

**Fig. 5:**
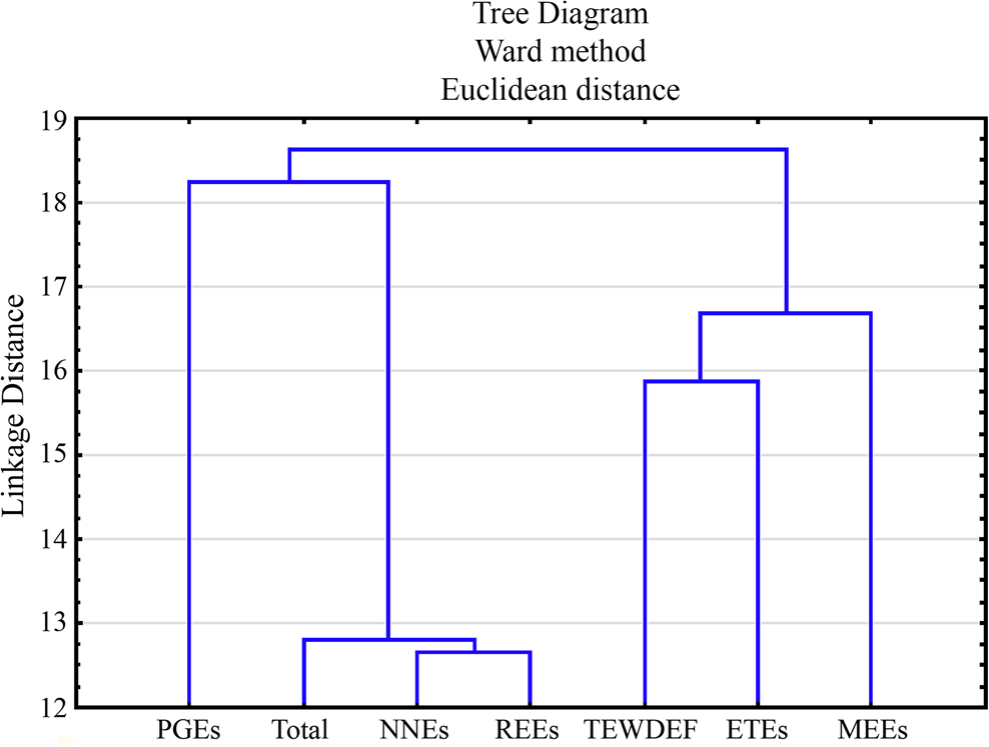
Cluster analysis calculated based on the rank sum to show similarities/differences between particular element groups determined in all 17 mushroom species jointly

Based on the rank sum described in Fig. 4 for mean content of particular groups of elements, the next rank sum was calculated and a cluster analysis was executed for six groups of elements. The Ward method allowed similarities to be shown between particular groups of elements (Fig. 5). The greatest similarity was observed between NNEs and REEs and also TEWDEF and ETEs.

According to the calculated Spearman rank correlation coefficients (*r*_s_), significant positive correlations were determined (*α* = 0.05) between NNEs and REEs (*r*_s_ = 0.8139), REEs and total (mean rank sum for all element groups jointly) (*r*_s_ = 0.7745), NEEs and total (*r*_s_ = 0.7990), TEWDEF and ETEs (*r*_s_ = 0.6906) and also MEEs and ETEs (*r*_s_ = 0.6577). Lower but also significantly positive correlations between PGEs and total (*r*_s_ = 0.5914), TEWDEF and total (*r*_s_ = 0.5739), REEs and PGEs (*r*_s_ = 0.5546) and also NNEs and PGEs (*r*_s_ = 0.5509) were also calculated. No further significant correlations were observed, which suggests that accumulation of mainly MEEs, ETEs and TEWDEF in the studied mushroom species is not correlated with REEs, NNEs or PGEs.

## Discussion

Interest in the mineral composition of fruit bodies of mushrooms, primarily edible, started in the 1970s and is ongoing. Hundreds of original papers on the topic have been published during the period. The main research results obtained during the individual phases have been collected in three reviews (Kalač and Svoboda 2000; Kalač 2010; Falandysz and Borovička 2013) and in a book (Kalač 2019). These publications collate data from over 700 original papers. The results given in this article will therefore be assessed in the light of the overall data collated chiefly in the book.

### Element contents

As results from extensive data for several widely consumed and analysed wild-growing species (Kalač, 2019), contents of both major and particularly trace elements within a species vary widely, commonly in order of magnitude. Such variability is notably higher than that found in vegetables and other crops.

As early as the 1980s, great differences were reported in the uptake of individual metals from underlying substrates (Tyler 1982; Gast et al. 1988). Substrate composition and acidity are important factors. The ability to accumulate an element from substrate to a fruit body is expressed by the bioaccumulation (or transfer) factor, the ratio of an element content in a fruit body to its content in the underlying substrate, both values in dry weight. If the value is > 1, an element is bioaccumulated, at value < 1, it is bioexcluded. Cadmium, mercury and copper are known to be accumulated in fruit bodies, levels of zinc and manganese are comparable in fruit bodies and in the relevant substrate, while contents of lead and iron are lower in fruit bodies than in the substrates.

Mycelium of saprobic species (e.g. *C. comatus*, *M. procera* or *P. gilva*), taking up nutrients from organic matter, is generally located in the litter layers, usually at or very close to the substrate surface. On the contrary, mycelium of mycorrhizal species is dispersed in the mineral layers where roots of the host plant are growing, i.e. at lower horizons.

Age of fruit body and its size have been assessed of low importance for element contents. Nevertheless, a recent report of Falandysz et al. (2020) brings an amendatory view. Results from their study with *Amanita muscaria* show that the contents of nutritionally essential K, Mg, Mn, Ni, Co, Cu, Zn and Se in fruit bodies remained throughout all developmental stages, the contents of Pb, Sb, Tl, Ba, Sr, Li, Rb and Cs decreased with increasing maturity, whereas V, Cr, As, Ag, Cd and U remained at the same level, similarly to the essential elements.

The proportion of an element originating from atmospheric depositions seems to be limited due to the short lifetime of fruit bodies of most aboveground species. There is a hypothesis (Kalač 2010) that the increasing age of mycelium, up to decades in wild-growing species, and a protracted interval between fructifications considerably elevate the contents of many elements in fruit bodies. Moreover, as results from available data show, most individual elements are distributed unevenly in the fruit body. Higher levels generally occur in caps than in stipes (Kalač 2019). Nevertheless, the situation is not unambiguous as may be seen for mercury when considering all the available information. Overall, laboratory data with many reports on mercury in mushrooms argue for the general opinion (Falandysz et al. 2015). Alonso et al. (2000) and Árvay et al. (2015) reported a higher level of mercury in the spore-forming part (hymenophore) than in the rest of fruiting body while no statistically significant differences were observed Melgar et al. (2009). Therefore, in our study, complete fruit bodies, as usually consumed, were samples.

Sixty-three determined mineral elements were classified into six groups. Four of them were constituted from major and trace elements of established nutritional or toxicological properties, and a further two groups from trace elements of a similar chemical nature (REEs and PGEs). From the 17 analysed species, original data were obtained for *L. cristata*, *L. personata, P. gilva* and *S. rugosoannulata* and for many elements in the other species.

Typical contents of MEEs in edible mushrooms reported during 2010–2018 (overall Kalač 2019) were as follows: 50–750, < 10,000–35,000, < 500–1500, 50–750 and < 2500–> 10,000 mg kg^−1^ (all contents are expressed per dry weight) of Ca, K, Mg, Na and P, respectively. The results of Table 1 are thus within the ranges of available data. Among the 17 analysed species, saprobic *C. comatus* and *P. gilva* from different families showed the highest total content of MEEs due to high levels of both K and P.

The contents of eight of the ten determined essential trace elements (ETEs) (Table 3) are also within the usual ranges of available data (Kalač 2019) being < 1–20, < 10–75, < 50–> 1000, < 25–100, < 0.5–2, 0.5–10, < 0.5–10 and < 25–200 mg kg^−1^ for B, Cu, Fe, Mn, Mo, Ni, Se and Zn, respectively. *M. procera* is known to be a copper accumulator. The contents of Co and Cr, mostly below limits of detection, are lower than those given in the literature, < 0.2–5 and < 0.5–20 mg kg^−1^, respectively.

The usual ranges of literature data (Kalač 2019) for the elements of the TEWDEFs group are < 0.5–5, < 0.5–10, < 0.2–10, < 1–5, < 0.5–5 and < 1–5 mg kg^−1^ for Ag, As, Ba, Cd, Hg and Pb, respectively. *A. arvensis* has been known to accumulate silver, *L. deliciosus* arsenic and *M. procera* mercury and cadmium. Generally, our results in Table 4 are similar to those of the two preceding groups, within the reported ranges. *A. arvensis* accumulated Ag to the greatest extent followed by *P. gilva*, which also contained the highest level of Ba. *L. deliciosus* showed the highest contents of Cd and Pb, and *M. procera* proved its ability to accumulate Hg. There is very limited literature data for a Tl range between < 0.1 and 0.3 mg kg^−1^, contents above 1 mg kg^−1^ in five species are thus surprising. No less likely cause could be the origin of selected fruit bodies growing on soils enriched in V, because even in the vicinity of Poznań, the concentration of this metal in soils shows high point anomalies (Lis and Pasieczna 2005). Beryllium was detected in only three species. This accords with very limited available data reporting below 0.1 mg kg^−1^ (notably Seeger et al. 1984). No toxicological relevance of Be in the analysed species can be thus supposed.

Literature data for rare earth elements (REEs) have been scarce. The levels of individual REEs are commonly < 0.1 mg kg^−1^ and seldom exceed 1 mg kg^−1^. Cerium, La and Nd were reported to occur in the highest contents (Kalač 2019; Siwulski et al. 2020). As can be seen in the results from Table 5, a different order was observed: Pr > Nd > La > Ho. *L. cristata* and *M. esculenta* are species with the highest total level of the REEs. The observed results enhance the knowledge of REEs in mushrooms. Overall, the contents of potentially detrimental REEs in mushrooms are low. The dietary intake from mushroom meals thus seems to pose no health risk.

Nineteen nutritionally non-essential trace elements (NNEs) form a group with generally limited literature data, apart from Rb and Al. Nevertheless, the contents of Al can be overestimated if mushroom samples are polluted with soil residues. Usual reported contents (Kalač 2019) are < 25–> 500, < 0.75, < 25–500, < 5, < 3, < 10, < 2, < 2 and < 1 mg kg^−1^ for Al, Li, Rb, Sr, Te, Ti, U, V and Zr, respectively. The determined contents in Table 6 fit well with the available data. The level of Sr in *M. esculenta* and those of Te in *A. arvensis*, *L. scabrum* and *L. aurantiacum* somewhat surpass limited existing knowledge. Literature data for Bi, Ga, Ge, In, Re and Sc have been very scarce and the determined contents in Table 6 present a contribution to the knowledge of these elements in mushrooms. Indium contents are higher than those of other elements in this subgroup. The contents of Au, Hf, Sb and Th were, with only several exceptions, below the detection limits. Interest in health detrimental platinum group elements (PGEs), particularly Pt and Pd, has increased since their use as vehicular converters has spread them into the environment. Existing data for mushrooms have been entirely insufficient. Table 7 thus purveys original values. Platinum is the element with the highest level within the group with a mean value of 5.0 mg kg^−1^ and contents above 10 mg kg^−1^ in two edible species, *M. esculenta* and *P. gilva*. Likewise, Rh was detected in all the analysed species, however, at a lower level, up to 0.79 mg kg^−1^ in *L. cristata.* This species also had the highest contents of Ir, Os, Pd and Ru. Generally, Ru, Pd and Ir were detected in only 1–2 of the analysed species. Overall, all the determined element contents in this work range within levels available in the literature (Kalač 2019).

### Role of species/family

There is a consensus that element contents in fruit bodies are species-dependent. Genus-dependence was sometimes stated, although with limited conclusiveness. Differences were observed even within a genus. A species of the genus *Agaricus* has been known to accumulate Cd, with a higher level of species yellowing (*flavescentes*) after mechanical damage of tissue (e.g. *A. arvensis*) than in those becoming red (*rubescentes*) (Andersen et al. 1982).

The accumulating ability of various families has been reported only to a limited extent. Pioneering works of the laboratory of Prof. Ruth Seeger from the University of Würzburg, Germany, should be underlined here. They analysed over 1000 samples of more than 400 edible, inedible and toxic species of numerous families and specified families with high and low abilities to accumulate four MEEs. However, the results were not then evaluated statistically. A low level of calcium was observed in the families Russulaceae and Lycoperdaceae (Seeger and Hüttner 1981). Potassium has been known as a quantitatively highly prevailing element in mushrooms. Seeger (1978) reported the highest contents in the former family Coprinaceae (recently Agaricaceae), including *C. comatus.* This species also showed the highest level of potassium within our set. The former family Coprinaceae was a high accumulator of magnesium, while the Boletaceae family was found to be the opposite ((Seeger and Beckert 1979). The data of Table 2 seem to confirm such a conclusion. Sodium was also accumulated mainly in the former family Coprinaceae (Seeger et al. 1983). Within our set, however, *B. edulis* contained significantly more sodium than *C. comatus* (463 and 286 mg kg^−1^, respectively).

Among trace elements, mercury was reported to be clearly species-dependent. Mercury-rich species were found particularly in Tricholomataceae, Amanitaceae and Lycoperdaceae, whereas rarely in Boletaceae, Amanitaceae and Russulaceae (Seeger 1976). However, later articles have reported high levels of mercury in *B. edulis* and *B. pinophilus* (Kalač 2010, 2019). *M. procera* from the family Agaricaceae, known as an Hg accumulator, contained noticeably the highest level among the tested species (Table 4).

According to available literature data, no further articles have reported mushroom families as determinants of mineral composition of fruit bodies. Nevertheless, consensus of species-dependency has developed on evidence based on a comparison of the element contents among various species. This article is the first to use recent statistical methods to assess the role of mushroom species/families in multielemental composition of fruit bodies.

PCA analysis allowed the high similarity in the content of elements belonging to particular groups to be shown (with the exception of MEEs) between species of the family Boletaceae (*B. edulis*, *L. aurantiacum* and *L. scabrum*), which may suggest that the mushroom family has a dominant role in the modification of the mineral composition of fruit bodies (Fig. 1). On the other hand, differences between mushroom species belonging to the family Agaricaceae observed for MEEs, ETEs and TEWDEF suggest that mushroom species may be a more important determinant of mineral composition of the fruit body. It is worth underlining that differences between species belonging to the same Tricholomataceae family were also observed (especially *T. equestre* in relation to the rest of the Tricholomataceae species as regards the content of MEEs and ETEs (Fig. 1a and b)), thus confirming the above-mentioned opinion.

As results from the heatmap (Fig. 2) show, from the five similarities mentioned in the “Similarities and differences between mushroom species” section, only one of them was between two species of the same family—*B. edulis* and *L. scabrum* (Boletaceae). Within seven dendrograms, only a low number of groups formed by species of the same family occur, namely *L. nuda* and *P. gilva* (Tricholomataceae) for MEEs and total elements (Fig. 3a, g), *L. nuda* and *T. equestre* (Tricholomataceae) for ETEs (Fig. 3b) and *L. aurantiacum* and *L. scabrum* (Boletaceae) for REEs (Fig. 3d). All other groups consist of species belonging to different families. All the described similarities/differences between species belonging to the same mushroom family are also partially confirmed by the sum rank (Fig. 4).

Overall, statistical evaluation of the comprehensive set of 63 mineral elements determined in 17 mushroom species belonging to 10 families revealed that the mineral contents in fruit bodies are primarily species-dependent, while family-dependency is of limited importance. The results thus endorse the rooted consensus.

## Conclusion

Unfortunately, a simple answer to the question of which taxonomic unit is the more important determinant of the mineral composition of fruiting bodies of mushrooms is not possible. Acquiring a significant number of fruiting bodies of different species of fungi belonging to the same but different families, coming from different surfaces, made it possible to answer this question. It is certain that species belonging to the same family may exhibit higher, lower or differentiated accumulation of individual elements. However, due to the differences in the content of elements included in particular groups of elements, the selective accumulation of individual elements and the differences found for species belonging to the Agaricaceae and Tricholomataceae families, it can be said with a high degree of probability that a mushroom species but not the family to which it belongs is a more important determinant of the mineral composition of the fruit body. It should also be emphasised that perhaps even more extensive research and more samples could confirm our observations or even definitively answer the question.
